# Real-time interfacial electron dynamics revealed through temporal correlations in x-ray photoelectron spectroscopy

**DOI:** 10.1063/4.0000099

**Published:** 2021-07-08

**Authors:** Felix Brausse, Mario Borgwardt, Johannes Mahl, Matthew Fraund, Friedrich Roth, Monika Blum, Wolfgang Eberhardt, Oliver Gessner

**Affiliations:** 1 Chemical Sciences Division, Lawrence Berkeley National Laboratory, Berkeley, California 94720, USA; 2 Physics Department, Universität Hamburg, 22607 Hamburg, Germany; 3 Institute of Experimental Physics, TU Bergakademie Freiberg, 09599 Freiberg, Germany; 4 Advanced Light Source, Lawrence Berkeley National Laboratory, Berkeley, California 94720, USA; 5 Center for Free-Electron Laser Science DESY, 22607 Hamburg, Germany

## Abstract

We present a novel technique to monitor dynamics in interfacial systems through temporal correlations in x-ray photoelectron spectroscopy (XPS) signals. To date, the vast majority of time-resolved x-ray spectroscopy techniques rely on pump–probe schemes, in which the sample is excited out of equilibrium by a pump pulse, and the subsequent dynamics are monitored by probe pulses arriving at a series of well-defined delays relative to the excitation. By definition, this approach is restricted to processes that can either directly or indirectly be initiated by light. It cannot access spontaneous dynamics or the microscopic fluctuations of ensembles in chemical or thermal equilibrium. Enabling this capability requires measurements to be performed in real (laboratory) time with high temporal resolution and, ultimately, without the need for a well-defined trigger event. The time-correlation XPS technique presented here is a first step toward this goal. The correlation-based technique is implemented by extending an existing optical-laser pump/multiple x-ray probe setup by the capability to record the kinetic energy and absolute time of arrival of every detected photoelectron. The method is benchmarked by monitoring energy-dependent, periodic signal modulations in a prototypical time-resolved XPS experiment on photoinduced surface-photovoltage dynamics in silicon, using both conventional pump–probe data acquisition, and the new technique based on laboratory time. The two measurements lead to the same result. The findings provide a critical milestone toward the overarching goal of studying equilibrium dynamics at surfaces and interfaces through time correlation-based XPS measurements.

## I. INTRODUCTION

The development of time-resolved spectroscopy^1^ has been driven by the goal to study elementary processes in atoms and molecules on their natural timescales. Most studies in this field are enabled through the use of the so-called pump–probe schemes. A pump laser pulse excites the system out of equilibrium, while a probe pulse, with variable and precisely adjustable delay, interrogates the sample evolution over time. The pump–probe scheme is an extremely powerful technique to resolve dynamics in systems ranging from isolated atoms to extended biological complexes. However, it inherently requires the dynamics to be initiated either directly or indirectly by an optical excitation. While many reactions can be triggered by light, the vast majority of chemical transformations occurring, for example, in living organisms or during chemical synthesis proceed through thermal activation of molecules in their electronic ground states.

The correlation spectroscopy (CS) approach provides a possible pathway to reveal such chemical dynamics.^2,3^ While time-averaged properties of a system in chemical or thermal equilibrium, such as, for example, the density of molecules on a sample surface, are constant in time, they fluctuate around their mean values on microscopic length scales and on timescales that are defined by reaction rates and molecular mobilities. Reaction rates themselves are time-invariant properties and can be determined through temporal correlations of spectroscopic signals,^4^ which are measured as a function of absolute laboratory time. A particularly prominent example for this approach is fluorescence correlation spectroscopy (FCS),^5^ which exploits temporal correlations in laser-induced fluorescence signals. This technique has been used to measure diffusion constants of the thermal motion of molecules and reaction rates in chemical equilibrium.^2,3^

To the best of our knowledge, only one example has been reported for translating FCS into the x-ray domain. In a proof-of-principle X-FCS experiment, Wang *et al.* used hard x-ray FCS to monitor diffusion and sedimentation dynamics of colloidal particles suspended in water.^6^ In the x-ray scattering domain, x-ray photon correlation spectroscopy (XPCS)^7^ is routinely employed to monitor spontaneous dynamics. There are, however, important distinctions. While FCS exploits number-density fluctuations in a small sample volume, which can be probed by incoherent spectroscopic signals, XPCS is based on the formation of speckles by interference of x-rays that propagate through the sample on different paths but reach the detector at the same *q*-vector. Fluctuations in the speckle pattern enable XPCS to probe random fluctuations in the distribution of scatterers in a material. By definition, the speckle phenomenon requires coherent x-ray flux, which does not apply to FCS or the technique presented here.

Translating CS to a larger variety of inner-shell spectroscopy techniques, such as x-ray photoelectron spectroscopy (XPS), offers a number of opportunities to combine the unique strengths of element specificity and chemical sensitivity of x-ray transitions with the dynamic insight provided by CS. For example, the excellent energy resolution of XPS can be exploited to distinguish molecular species that are chemically very similar, such as molecules chemisorbed on different surface sites or molecules that are part of an ensemble undergoing an isomerization reaction, but sufficiently different to affect the local chemical environment of a particular element, leading to a distinct shift in core-level binding energies. This sensitivity is routinely exploited in electron spectroscopy for chemical analysis (ESCA).^8,9^ Another opportunity provided by the detection of photoelectrons lies in the extreme surface sensitivity of XPS. In combination with ambient-pressure capabilities, one may envision, for example, the microscopic real-time study of interfacial equilibrium dynamics between gases or liquids and a solid bulk catalyst by time-correlation XPS (TCXPS).

Here, we present an implementation of TCXPS based on the event-by-event recording of laboratory-time photoelectron data that include the absolute time of arrival and the kinetic energy of every single photoelectron, from which temporal and spectral correlations are calculated after the measurement. The test experiment monitors photoinduced surface photovoltage (SPV) dynamics in *p*-doped silicon^10^ using a laser-pump/x-ray-multiple-probe setup that is able to measure both conventional pump–probe traces and laboratory-time XPS event streams. The test experiment focuses on photoinduced, non-equilibrium dynamics in order to provide a rigorous test of the correlation-based analysis. The recording of pump–probe and correlation data with the same setup back-to-back under virtually identical conditions provides the most stringent, quantitative test of the correlation approach. To demonstrate the equivalence between both methods, relaxation dynamics derived from the correlation data using basic model assumptions are compared to the dynamics observed in pump–probe mode. We note that the use of a reproducible, periodic excitation by the pump laser simplifies the correlation analysis and is only a first step toward future TCXPS studies of spontaneous chemical dynamics, the feasibility of which still needs to be demonstrated. Yet, the presented results establish important milestones toward this long-term goal under well-controlled conditions that are more challenging to achieve for spontaneous processes.

## II. EXPERIMENT

**FIG. 1.**
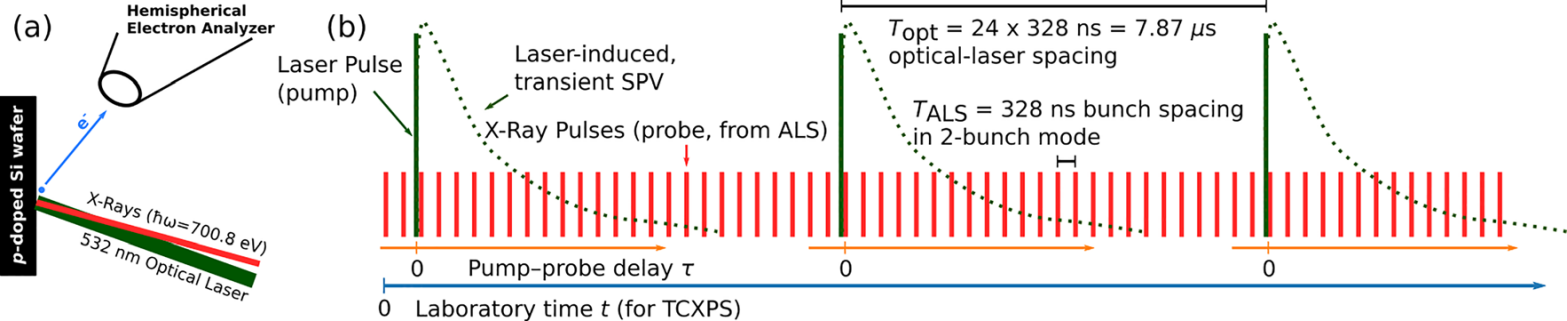
(a) Sketch of the experiment. (b) Temporal structure of the optical laser (tall vertical bars) and x-ray pulse trains (red bars). The dotted green traces schematically illustrate the periodic sample response to the laser excitation. Orange and blue horizontal arrows at the bottom of the panel indicate the two different reference systems for measuring time. In the pump–probe experiment (orange), time is referenced to the pump laser pulses with a maximum timescale corresponding to the spacing between two laser pulses. The correlation experiments (blue) operate in the laboratory timeframe with an arbitrary chosen time zero and a maximum timescale only limited by the maximum sampling range of the detector electronics.

The time-resolved x-ray photoelectron spectroscopy (tr-XPS) setup used in the experiment has previously been described in detail in Refs. 11 and 12. Briefly, photoelectrons generated on the sample surface are detected in a hemispherical electron analyzer that translates the photoelectron kinetic energy into a specific impact position on the detector. A fast delay-line detector is used to register the time and position of each electron impact. In the present experiment, a soft x-ray beam (ℏω = 700.8 eV) from beamline 11.0.2 of the Advanced Light Source (ALS) is spatially overlapped with an optical laser beam (wavelength λ = 532 nm, fluence 0.49 mJ/cm^2^) on a *p*-doped (100) silicon wafer (resistivity ρ ≈ 3.5 Ω cm), as sketched in Fig. 1(a). The optical pump pulses induce above-bandgap excitations in the Si substrate, leading to transient variations in the electronic band bending toward the surface, that is, a time-dependent SPV, which is reflected in transient binding energy shifts of the Si 2*p* photolines.^10^ The ≈127 kHz repetition rate of the optical laser is chosen such that one laser pulse is generated for every 24 x-ray pulses [Fig. 1(b)], with the ALS operating in two-bunch mode at an x-ray pulse spacing of 328 ns.

Data are acquired in three distinct modes, the pump–probe mode and two TCXPS modes. In the pump–probe mode, the optical laser and the detector are synchronized to the ALS master clock, which we refer to as “fully synchronized.” In the TCXPS modes, the detector hardware is not synchronized to either the optical laser or the synchrotron pulses. Therefore, measurements in the traditional pump–probe mode and in the TCXPS modes have to be taken separately. In one TCXPS mode, the optical laser and the ALS master clock are synchronized to each other to establish a constant phase relation between the two. The relative pulse timing is identical to the pump–probe experiment as shown in Fig. 1(b). Therefore, this operating mode is referred to as a “phase-stabilized” TCXPS. In the other case, the phase lock between the laser and the synchrotron is lifted, causing the laser pulse timing to drift relative to the x-ray pulses. In this case, all possible pump–probe delays are sampled over the duration of the experiment, not only the 24 points sketched in Fig. 1(b). This operating mode is referred to as “free-running” TCXPS. In the fully synchronized (pump–probe) and the phase-stabilized TCXPS modes, the delay between the optical laser pulses and the x-ray pulses is fixed such that the laser pump pulse arrives ∼800 ps before the next x-ray pulse.

In all experiments, the hemispherical analyzer is operated with constant voltages at a photoelectron kinetic energy of *E*_kin_ = 600 eV and a pass energy of *E*_pass_ = 30 eV, focusing on the Si 2*p* photolines with ∼99.6 eV binding energy. Ahead of every data acquisition run, which typically spans 900 s, the silicon wafer is irradiated with a laser fluence of ∼30 mJ/cm^2^ for 120 s to remove the native SiO_2_ layer. Additional XPS reference measurements with the optical laser turned off are referred to as steady-state experiments in the following.

Conventional pump–probe spectra are generated by collecting histograms of the detected photoelectrons inside the detector hardware, yielding two-dimensional data sets of detector position (i.e., kinetic energies) vs time-of-arrival relative to the laser trigger, from which the pump–probe delay τ [Fig. 1(b)] can be calculated. With the new TCXPS method implemented here, the time of arrival *t_i_* and impact position/kinetic energy *e_i_* of each *i*th detected photoelectron is stored separately, and *t_i_* is measured in the laboratory frame; that is, it is referenced to the beginning of the data acquisition period, not the laser trigger. The resulting datasets consist of event tables with *N* lines for *N* detected photoelectrons.

## III. DATA ANALYSIS

**FIG. 2.**
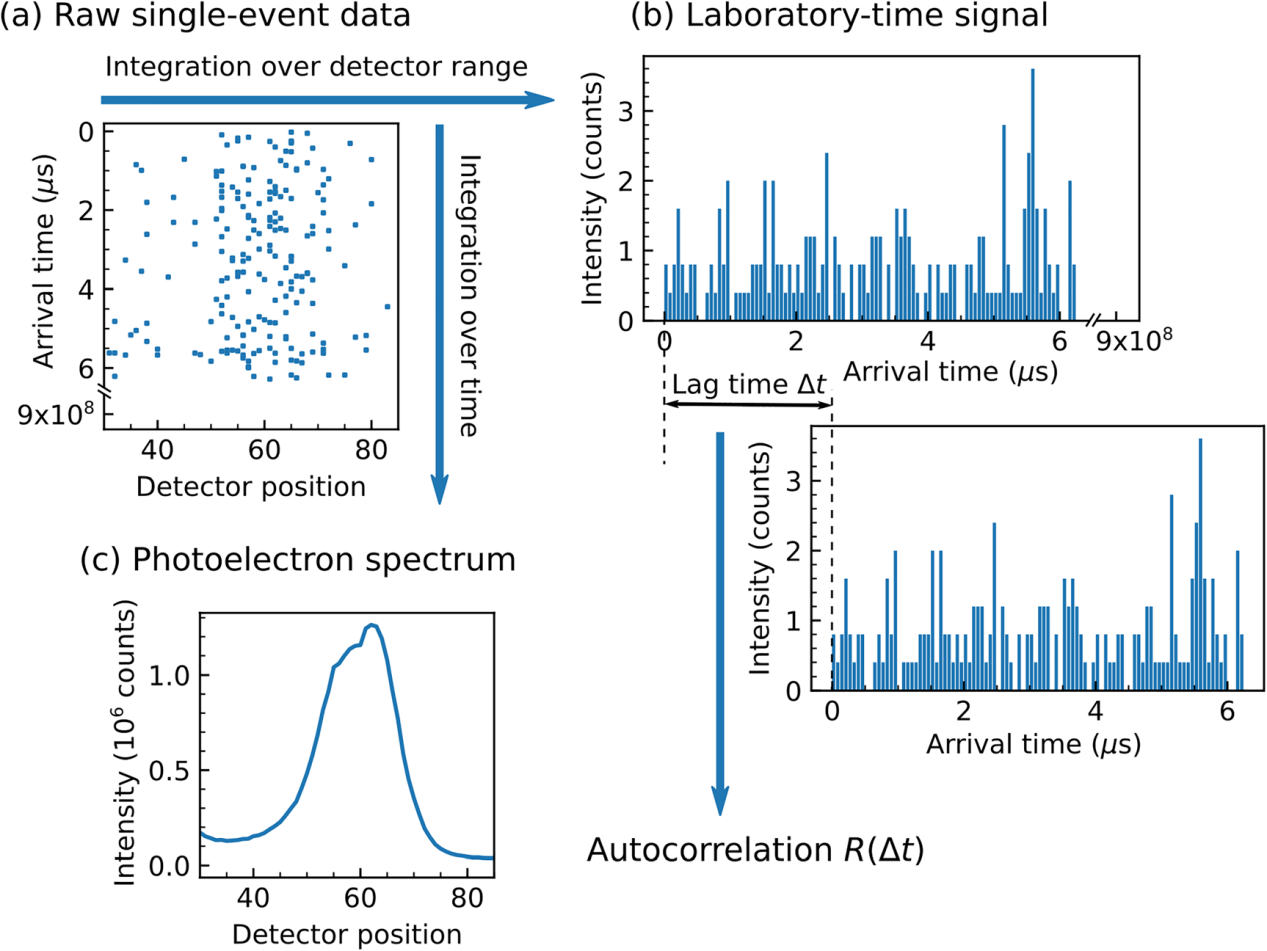
Illustration of the single-event TCXPS data structure and derived quantities. (a) Recorded pairs of arrival times *t_i_* and detector positions *e_i_*, shown as individual points. (b) Integration over a range of detector positions yields laboratory time-dependent electron signals, which form the basis of the temporal correlation analysis. (c) Integration over all arrival times yields the conventional, time-averaged photoelectron spectrum. Note that the data in panel (a) only show the first 200 out of ∼10^7^ events contained in the spectrum in panel (c).

The nature of the single-event data is illustrated in Fig. 2(a), where (*t_i_*, *e_i_*) pairs are shown as individual points. This format represents the maximum information content of the data, which are further processed to access various observables and their temporal correlations. For instance, integration of Fig. 2(a) over a range of detector positions yields the laboratory time-dependent total electron signal within the corresponding energy range [Fig. 2(b)]. Similarly, integration over all arrival times yields the conventional, time-averaged photoelectron spectrum [Fig. 2(c)].

The temporal autocorrelation of a time- and energy-dependent signal *f*(*e*, *t*) may be illustrated as the overlap of the signal with a time-delayed copy *f*(*e, t*–Δ*t*) [Fig. 2(b)]. In general, this overlap may be evaluated for any two kinetic energies, *e*_1_ and *e*_2_, resulting in a correlation function, *R*(*e*_1_, *e*_2_, Δ*t*), which depends on three variables: the arrival time difference (“lag time”) Δ*t*, and the energies *e*_1_ and *e*_2_. If *e*_1_ = *e*_2_, we refer to *R* as an autocorrelation function and otherwise as a cross correlation between signals at energies *e*_1_ and *e*_2_. Here, *f*(*e*, *t*) is measured on a discrete, equidistant grid in both kinetic energy (through the detector positions) and laboratory time (through the temporal resolution of the detector, here defined as *w*). Positions along the time axis can be described by integer multiples of the detector resolution: *t_m_* = [0*w*, 1*w*, 2*w*, … *mw*, (*m *+* *1)*w*, …]. Thus, the lag time can be written as Δ*t* = *mw* and *f*(*e*, *t*) can be reduced to *f_m_*(*e*). If the intervals span from *m *=* *0 to some maximum *m *=* M*, the correlation is given by^13^

$$R\left(e_{1},\;e_{2},\;\Delta t=kw\right)=\frac{1}{\left(M-k\right)}\sum_{m=0}^{M-k}\frac{f_{m}\left(e_{1}\right)f_{m+k}\left(e_{2}\right)}{\left\langle f\left(e_{1}\right)\right\rangle \left\langle f\left(e_{2}\right)\right\rangle },$$ (1)

where ⟨ *f* (*e*)⟩ is the average of *f_m_*(*e*) over all *m*. For computational efficiency, correlation functions are calculated from the photoelectron event data by using a sparse correlation algorithm as described in the supplementary material.^18^

## IV. RESULTS AND DISCUSSION

### A. Steady-state correlation experiment

**FIG. 3.**
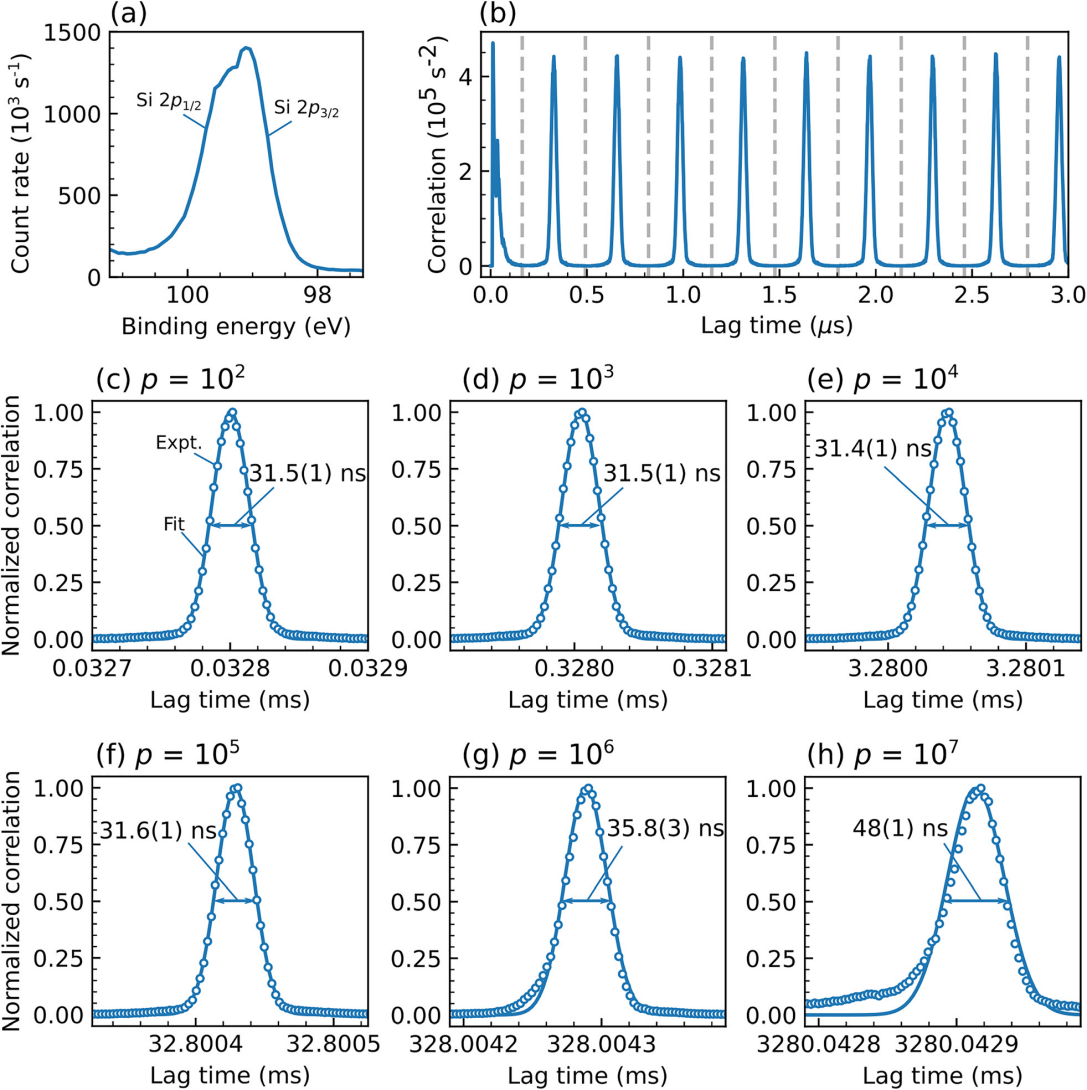
Steady-state TCXPS experiment without optical excitation. (a) Si 2*p* photoelectron spectrum of a *p*-doped Si (100) substrate. The two spin–orbit components are not resolved but indicated by the peak asymmetry. (b) Temporal autocorrelation function of the signal in (a), integrated over the entire kinetic energy range. Vertical dashed lines indicate the integration regions used to determine the total area under the correlation peaks (see the text for details). (c)–(h) Details of the TCXPS peaks (circles) and fits to a Gaussian function (solid line) for lag times Δ*t* = *p*·328 ns with *p *=* *10^2^ (c), *p *=* *10^3^ (d), *p *=* *10^4^ (e), *p *=* *10^5^ (f), *p *=* *10^6^ (g), and *p *=* *10^7^ (h). Double-headed arrows indicate the full width at half-maximum (FWHM) of the Gaussian fit results.

Before discussing the TCXPS results for laser-induced dynamics, it is instructive to analyze data recorded in steady-state mode, that is, with the optical laser off. Summation over all electron arrival times recovers the conventional Si 2*p* photoelectron spectrum [Fig. 3(a)]. Note that the two spin–orbit components Si 2*p*_1/2_ and Si 2*p*_3/2_ are not resolved but indicated by a notable asymmetry of the photoelectron peak. Integrating the signal over the entire detector and calculating the temporal autocorrelation of this energy-integrated signal reveals the periodic trace shown in Fig. 3(b). The period of this signal corresponds to the pulse-to-pulse spacing *T*_ALS_ = 328 ns of the ALS in two-bunch operating mode. The periodicity of the correlation function spans many orders of magnitude in lag time, as exemplified by the correlation peaks corresponding to 100 to 10^7^ ALS bunch periods, shown as blue circles in Figs. 3(c)–3(h).

The autocorrelation trace shown in Figs. 3(b)–3(h) can be qualitatively understood by recalling that the x-ray pulse duration of ∼70 ps is much shorter than the pulse-to-pulse spacing *T*_ALS_. Therefore, photoelectron emission occurs in short bursts during the interaction of the sample with the x-ray pulses, leading to a time-dependent photoemission signal that repeats every integer period *p* at a lag time of Δ*t* = *p*·*T*_ALS_. Up to ∼10^5^ periods, the shapes of the correlation peaks are virtually indistinguishable from each other, except for the very first one at a lag time of 0 [Fig. 3(b)]. This first peak contains information about the temporal distribution of the photoelectron arrival times from a single x-ray pulse on the detector, leading to some additional structure within the peak. As this information is not relevant for the sample response on ∼ns and longer timescales, we exclude the first correlation peak from the analysis. After ∼10^6^ periods, the correlation peak shape begins to broaden and an asymmetric pedestal starts to emerge [Fig. 3(g)].

A more quantitative analysis of the autocorrelation function is provided by fits to a Gaussian function, $G\left(\Delta t,\;\mu ,\sigma \right)=\frac{1}{\sigma \sqrt{2\pi }}\text{exp}(\frac{-{\left(\Delta t-\mu \right)}^{2}}{{2\sigma }^{2}})$, shown as solid lines in Figs. 3(c)–3(h). For the first four peaks [Figs. 3(c)–3(f)], the full width at half-maximum (FWHM) of 32 ns agrees within the precision of the fit (≈2%), and no indications for peak shape changes are observed within the experimental uncertainty. For the peaks in Figs. 3(g) and 3(h), the FWHM increases to 36 and 48 ns, respectively, and noticeable deviations from a Gaussian peak shape become apparent. Still, the observed dephasing, that is, the pedestal extending to short lag times in Fig. 3(h), is on the order ∼50 ns at a lag time of 3 s, which corresponds to a relative deviation smaller than 10^−7^. As all lag times correspond to time differences that are recorded at arbitrary absolute laboratory times, correlations in Fig. 3 for long lag times are sensitive to time-averaged, relative changes between the periodicity of the synchrotron pulses and the stability of the detector clock. The observed relative dephasing on the order of ≤10^−7^ is likely dominated by the detector timing, as typical quartz oscillator drifts are on this order of magnitude. The results suggest that the present implementation of the laboratory-time based data acquisition should enable correlation measurements spanning at least seven orders of magnitude in lag time.

In general, the time resolution of a correlation measurement is determined by three limiting factors: the repetition rate of the x-ray source, the temporal resolution of the spectrometer, and the temporal resolution of the detector. In the present experiment, the time resolution is determined by the 328 ns x-ray pulse spacing. Still, we note that the autocorrelation peak width of 32 ns is more than two orders of magnitude larger than the theoretical lower limit of 100 ps one may expect from the autocorrelation of 70 ps long x-ray pulses. The peak broadening is caused by the finite photoelectron time-of-flight (TOF) spread within the hemispherical analyzer, as discussed in detail in Ref. 11. In this work, it has been demonstrated that the TOF spread can be reduced to ≲1 ns by a different choice of electron kinetic energies and/or pass energies. In principle, this reduced TOF spread is sufficient to exploit the minimum x-ray pulse spacing of 2 ns of the ALS in multi-bunch operating mode for correlation experiments. With the current setup, however, the dead time of the delay-line detector (∼10 ns) would define the overall achievable temporal resolution. In practice, depending on the experimental conditions and sample characteristics, a compromise may have to be found between TOF spread and kinetic energy resolution. As the TOF-spread and, therefore, the correlation peak width is much larger than the x-ray pulse duration, every peak can be integrated over its full width in time without losing information relevant to the time-dependent sample response. Thus, in the following, for determining sample dynamics through the correlation analysis, signals are integrated over the lag time intervals indicated by the vertical dashed lines in Fig. 3(b).

### B. Pump–probe reference measurement

**FIG. 4.**
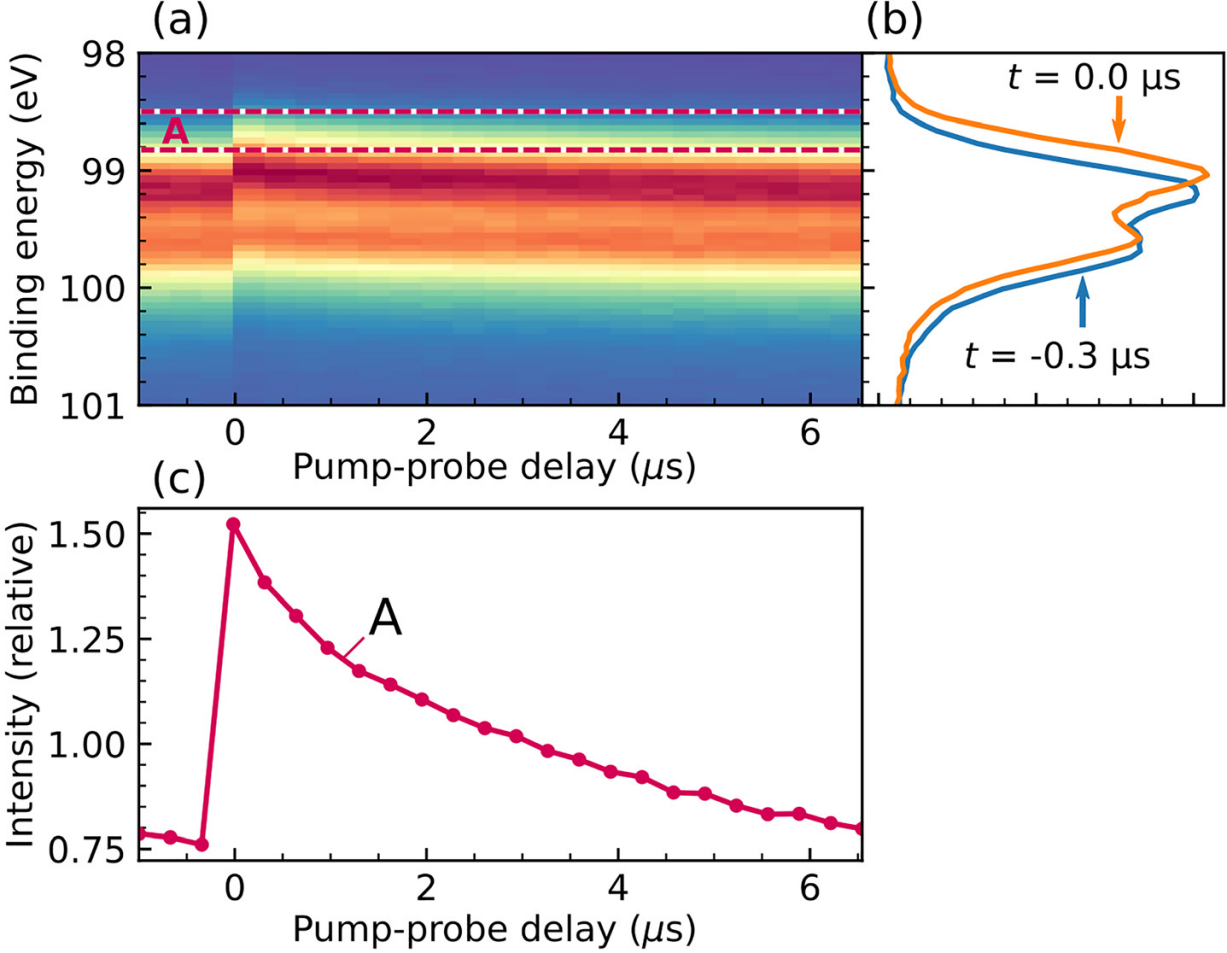
Pump–probe reference measurement. (a) Time-resolved Si 2*p* photoelectron spectrum of a *p*-doped silicon (100) sample, excited with 532 nm laser pulses. Dashed lines indicate the energy integration region A that is used to compare the pump–probe with the correlation-XPS analysis. (b) Photoelectron spectra recorded before (blue) and 800 ps after (orange) optical excitation. (c) Time-dependent signals obtained from integrating the time-resolved photoelectron spectra in (a) over energy region A. Intensities are normalized to signal averages over all pump–probe delays.

To quantify the SPV dynamics of the silicon sample, a reference measurement is carried out in the conventional pump–probe data acquisition mode. A typical time-resolved photoelectron spectrum is shown in Fig. 4(a). Upon sample excitation by the pump pulse, the entire spectrum shifts to smaller binding energies by approximately 120 meV. This SPV effect^14^ is caused by a photoinduced reduction of the intrinsic downward band-bending in *p*-doped silicon toward the surface.^10–12,15^ When a laser pulse induces superbandgap transitions in the semiconductor, mobile electron–hole pairs are created that can counteract the dipole fields underlying interfacial band bending, leading to a reduction of the downward band bending (“band flattening”) and a corresponding transient shift of the core levels to smaller binding energies. Through charge-carrier recombination, the SPV gradually decays on a microsecond timescale.

In Fig. 4(b), a photoelectron spectrum recorded before the arrival of the pump pulse (τ = −0.3*μ*s, blue) is compared to one recorded at the smallest positive pump–probe delay of +800 ps (τ = 0 *μ*s, orange), where the SPV effect is most pronounced. The rigid shift in binding energies leads to strong intensity modulations in spectral range A, which contains the low binding energy wing of the peak, in particular the region of greatest relative change in intensity. In the following, the intensity modulations in this region, shown as relative change in Fig. 4(c), are used to demonstrate the TCXPS analysis and to compare it with the results of a conventional pump–probe analysis. Note that the traces in Fig. 4(c) are normalized to the signal average over all pump–probe delays, which is most suitable for comparison with the TCXPS results.

### C. Reconstruction of dynamic trends from TCXPS data

**FIG. 5.**
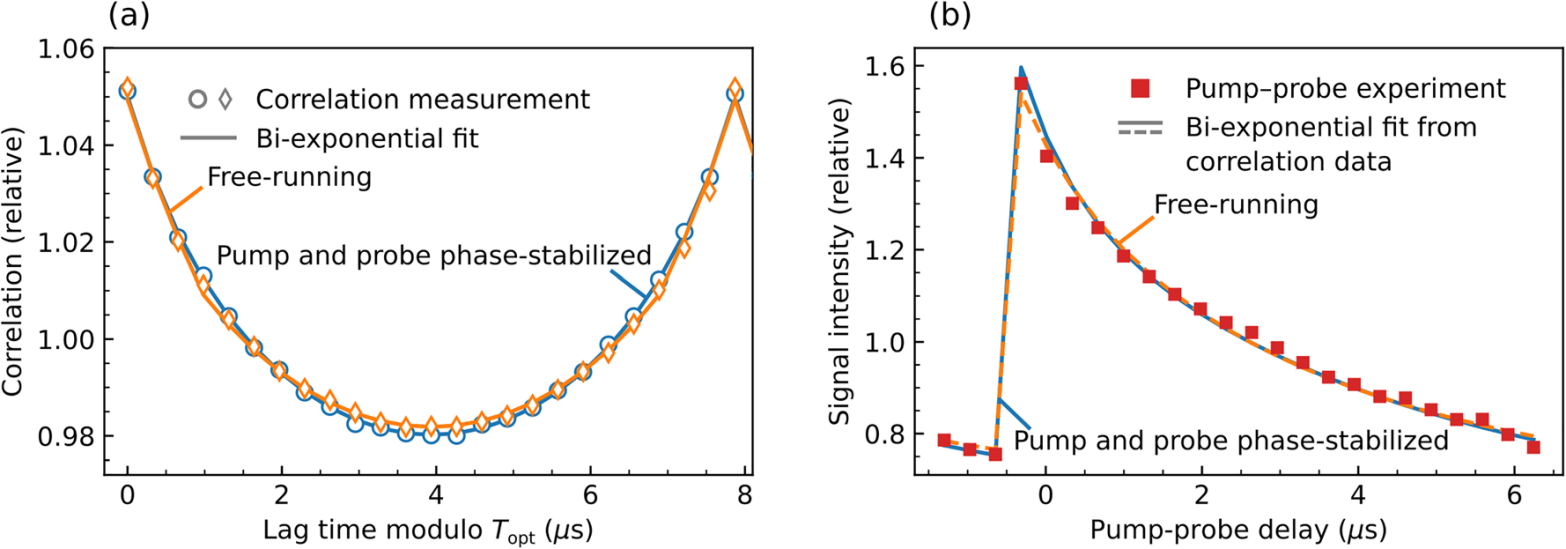
Reconstruction of SPV dynamics from the TCXPS experiment. (a) Cycle-averaged autocorrelation data (see the supplementary material for details^18^) for region A (circles and diamonds) compared to least squares fits that derive temporal autocorrelations from bi-exponential model functions of the SPV dynamics (solid lines). Blue and orange colors indicate results for data recorded under phase-stabilized and free-running laser/x-ray conditions, respectively. (b) Time-dependent intensity modulation in region A as determined by the pump–probe measurement (red squares) and as determined from the fits in panel (a) (blue and orange lines).

For the vast majority of dynamic phenomena, the actual temporal evolution of a system is of primary interest, while auto- or cross-correlations are a means to make inferences about the temporal evolution where it cannot be directly measured. Thus, it is of particular interest to assess, to which degree dynamic trends that are readily available from pump–probe experiments can be reconstructed from a TCXPS measurement. A universal, deterministic transformation from temporal correlations to real-time dynamic trends does not exist. However, basic assumptions regarding the dynamics can often provide sufficient boundary conditions to enable a unique reconstruction of dynamic trends within these model assumptions. Such an approach is illustrated in Fig. 5.

The symbols in Fig. 5(a) are autocorrelation data from region A [Fig. 4(a)], recorded in the phase-stabilized (blue circles) and free-running (orange diamonds) TCXPS modes. Note that the data shown in Fig. 5(a) correspond to an average over 1000 laser cycles spanning a total lag time range of ∼8 ms. The data were recorded over a data acquisition time of 1 h. Relative standard deviations of the autocorrelation function for lag times spanning a single laser cycle are typically on the order of ∼6 × 10^−2^, translating into a relative standard deviation of ∼10^−3^ for the cycle-averaged data. Details of the data analysis as well as autocorrelation traces before cycle-averaging are discussed in Sec. II of the supplementary material.^18^ Real-time dynamics are reconstructed based on the assumption that the SPV dynamics underlying the measured autocorrelation traces may be described by a bi-exponential decay model, which is frequently the case.^12^ Within this boundary condition, the autocorrelation traces in Fig. 5(a) can be modeled by five independent parameters: two decay time constants τ_i_, two amplitudes *A*_i_, and a constant offset *C*. A fit procedure for the measured autocorrelation traces is defined by applying a numerical autocorrelation to the bi-exponential SPV decay model function, and iteratively varying the five model parameters toward best agreement in a non-linear, least squares fit approach. Figure 5(a) shows the result of these fits as blue and orange lines compared to the autocorrelation traces for region A measured in phase-stabilized and free-running mode, respectively. We note that the modulation depth of the free-running data has been scaled by a factor *f*^2^, with *f *=* *0.84. This scaling compensates for an ∼16% difference in SPV amplitudes of data recorded on different days. The variation is likely the result of slightly different sample surface and laser excitation conditions. Sample aging and inhomogeneity effects, such as a varying thickness of the native silicon oxide layer that grows over time on the silicon surface, can significantly affect the SPV amplitude. Slight day-to-day variations in the laser intensity and focusing conditions may also be a contributing factor.

For the phase-stabilized TCXPS measurement, the fit of the autocorrelation trace in Fig. 5(a) leads to decay constants of τ_1_ = 1.3 ± 0.4 and τ_2_ = 13 ± 2 *μ*s for the fast and slow SPV relaxation components, respectively. For the measurement with the free-running TCXPS mode, these time constants are τ_1_ = 2.2 ± 0.8 and τ_2_ = 11 ± 3 *μ*s. The corresponding bi-exponential model functions are plotted as solid lines in Fig. 5(b) along with the results of the pump–probe measurement (red squares). As noted above, the results for the free-running data were scaled by *f *=* *0.84. Very good agreement is found between the dynamic trends measured directly by the conventional pump–probe approach and reconstructed from the correlation measurements in both phase-stabilized and free-running TCXPS modes. A direct fit of the pump–probe data with a bi-exponential model function leads to decay time constants of τ_1_ = 1.2 ± 0.2 and τ_2_ = 13.5 ± 1.2*μ*s. These timescales agree well with those reconstructed from the TCXPS data within the given uncertainty ranges.

The results demonstrate that, based on an educated guess, quantities like decay constants can be quantitatively recovered from temporal correlations. While some information, such as the relative phases between the fast and the slow components, is lost in the correlation measurement, the physics that underlie the processes under investigation often provide sufficient boundary conditions to compensate for this loss. Alternative comparisons between the pump–probe and the TCXPS results that concentrate on auto- and cross-correlations, rather than on the time-dependent trend itself, are described in Sec. II of the supplementary material.^18^

The 328 ns x-ray pulse spacing in the experiments described herein limits the temporal resolution of the correlation approach. Efforts are under way to employ the multi-bunch operating mode of the ALS with a 2 ns pulse spacing to significantly improve the temporal resolution. We note that, principally, the method should be extendable into the femtosecond regime using x-ray free electron lasers (XFELs) in combination with a recently developed correlation analysis technique that exploits ultrafast split-pulse schemes.^13,16^ While fast detectors can enable ∼*μ*s time-resolved XFEL measurements,^17^ to access even shorter timescales, signals from different x-ray pulses are not separated by their detection times, but the lag time is defined by a well-controlled double-pulse spacing and the sum of signals from both pulses is detected. As shown by Gutt *et al.*, the two schemes can provide equivalent information.^13^ The practical implementation of split-pulse XPCS at XFELs with improved temporal resolution has been demonstrated by Roy, Turner, and collaborators.^16^ We expect that a similar transfer of TCXPS to XFEL conditions should be possible, but also note that it will require careful shot-by-shot monitoring of intensity fluctuations in each of the x-ray pulses.

## V. CONCLUSIONS

A method is presented for extracting real-time dynamics in XPS experiments through temporal correlations in the detected photoelectron signals. The technique is enabled by the capability to separately record the kinetic energy and absolute arrival time of every single detected photoelectron. The successful implementation of the technique is demonstrated in a benchmark experiment on laser-induced transient SPV effects in a Si sample. Excellent agreement is found between the results of the TCXPS and pump–probe approaches. The correlation analysis involves contributions spanning up to seven orders of magnitude in lag-time, providing opportunities for future studies of processes involving a vast range of timescales, simultaneously monitored in a single TCXPS experiment. The demonstration experiment uses a periodic excitation by the pump laser, which enables direct comparison between TCXPS and pump–probe results and simplifies the correlation analysis. Future efforts will focus on developing TCXPS further into a tool for monitoring spontaneous dynamics as previously demonstrated for related techniques, such as FCS and XPCS. We envision a wide range of applications, such as the study of diffusion dynamics of molecules on liquid and solid surfaces, adsorption/desorption kinetics at gas–solid interfaces in and out of equilibrium, spontaneous chemical dynamics on catalyst surfaces, and spontaneous phase transitions.

## Data Availability

The data that support the findings of this study are available from the corresponding author upon reasonable request.
